# Prevalence of COVID-19 fear and its association with quality of life among fire service recruits after ceasing the dynamic zero-COVID policy in China

**DOI:** 10.3389/fpubh.2023.1257943

**Published:** 2023-10-06

**Authors:** Jian Liu, Tong Leong Si, Pan Chen, Yue-Ying Wang, Zhaohui Su, Teris Cheung, Todd Jackson, Yu-Tao Xiang, Yuan Feng

**Affiliations:** ^1^Department of Rehabilitation Medicine, China Emergency General Hospital, Beijing, China; ^2^Unit of Psychiatry, Department of Public Health and Medicinal Administration, Institute of Translational Medicine, Faculty of Health Sciences, University of Macau, Taipa, Macao SAR, China; ^3^Centre for Cognitive and Brain Sciences, University of Macau, Taipa, Macao SAR, China; ^4^School of Public Health, Southeast University, Nanjing, China; ^5^School of Nursing, Hong Kong Polytechnic University, Kowloon, Hong Kong SAR, China; ^6^Department of Psychology, University of Macau, Taipa, Macao SAR, China; ^7^Beijing Key Laboratory of Mental Disorders, National Clinical Research Center for Mental Disorders & National Center for Mental Disorders, Beijing Anding Hospital, Capital Medical University, Beijing, China

**Keywords:** COVID-19 pandemic, fire service recruits, fear of COVID-19, network analysis, quality of life

## Abstract

**Background:**

In December 2022, China terminated its dynamic zero-COVID policy. To date, however, no research has been conducted upon mental health issues and their relationship with quality of life (hereafter QoL) among fire service recruits since the dynamic zero-COVID policy ended. This study explored fear of COVID-19 (FOC) prevalence and correlates as well as its network structure and interconnections with QoL among fire service recruits.

**Methods:**

A cross-sectional survey design was used to assess fire service recruits in Beijing and Sichuan, Guangxi and Guizhou provinces of China between February 13 and 16, 2023. Fear of COVID-19 was measured using the Fear of COVID-19 Scale, depression was assessed with the Patient Health Questionnaire, anxiety was examined using the Generalized Anxiety Disorder scale, and QOL was evaluated with the World Health Organization Quality of Life-brief version. Univariate and multivariate analyses were used to explore correlates of COVID-19 fear. Network analysis assessed the structure of fear of COVID-19 and its associations with QoL.

**Results:**

A total of 1,560 participants were included in this study. The overall prevalence of fear of COVID-19 was 38.85% (*n* = 606; 95% CI = 36.42–41.32%). Being afraid of COVID-19 was significantly related to depression (OR = 1.084; *p* < O.OO1) and physical fatigue (OR = 1.063; *p* = 0.026). Fire service recruits with more fear of COVID-19 had lower QOL (*F* = 18.061 *p* < 0.001) than those with less fear of COVID-19 did. The most central symptoms included FOC6 (“Sleep difficulties caused by worry about COVID-19”), FOC7 (“Palpitations when thinking about COVID-19”) and FOC2 (“Uncomfortable to think about COVID-19”). The top three symptoms negatively associated with QoL were FOC4 (“Afraid of losing life because of COVID-19”), FOC6 (“Sleep difficulties caused by worry about COVID-19”) and FOC2 (“Uncomfortable to think about COVID-19”).

**Conclusion:**

Over one-third of fire service recruits reported fear of COVID-19 after China’s dynamic zero-COVID policy had terminated. Poorer QoL was related to fear of COVID-19. Targeting core symptoms of the fear network structure could help improve the physical and mental health of fire service recruits during public health crises.

## 1. Introduction

In late January 2020, the COVID-19 outbreak rapidly spread, causing global concern (1). China lifted its almost three-year dynamic zero-COVID policy in December 2022, leaving Omicron variants in wide circulation across the country (2). Thereafter, rapid spread of the virus led to increased mental health problems among many people due to fear of getting sick, social isolation and economic uncertainty as well as reduced quality of life (QoL) (3). In addition, lockdowns and other pandemic control measures have resulted in widespread job losses, business closures, and economic hardship for many individuals and communities (4).

Fire service recruits are individuals who receive specialized training to acquire skills and competencies required by the fire service (5). According to relevant regulations and policies in China, the national comprehensive fire and rescue team is open to the community and new entry firefighters are required to participate in a one-year induction training program. If recruits fail the training and assessment, they will be dismissed (6). Fire service recruits undergo technical and operational training as well as training in mental health and stress management (7). The mental health of firefighters has been subject to significant challenges during and following the pandemic. In response to the pandemic, the Chinese government implemented policies and measures to combat COVID-19. As a result, training for fire service recruits occurred within specific premises, from which they were not permitted to leave freely, increased risk of loneliness, depression and fear were possible consequences of this policy (8, 9). Therefore, understanding mental health problems of fire service recruits could help to reduce distress and promote mental well-being for this population.

Fear, an emotion associated with physiological arousal and emotional avoidance of specific stimuli, is linked to clinical phobias and anxiety disorders when expressed in extreme form (10). Previous research (11) estimated the prevalence of fear of COVID-19 was 18, 19, and 33.7% in general population samples from Bosnia and Herzegovina (12), Brazil (13), and Lebanon (14), respectively. Potential widespread public fear brought on by the COVID-19 pandemic could result in severe emotional distress and a variety of coping responses to perceived dangers of infectious disease (15). Moreover, chronic fear can be emotionally exhausting, leading to feelings of helplessness and hopelessness that are characteristic of depression (16, 17). Prolonged activation of stress responses associated with fear and anxiety can also contribute to disruptions in the regulation of stress hormones and affect brain regions involved in mood regulation (18). Other research has found significant associations between higher fear of COVID-19 levels and perceived job insecurity, decreased job satisfaction and poor quality of life (QoL) (19).

Network analysis has emerged as a novel approach to conceptualizing psychological phenomena including fear of COVID-19 (20). Core concepts of network analysis include nodes, that reflect individual symptoms, and edges that represent associations between symptoms (21). Based on network theory, psychological disorders or syndromes can be viewed as networks of interacting symptoms (20). Network models can help to elucidate the most central nodes in a network structure as well as the edges that reflect the strongest relationships between symptoms; such central symptoms serve as plausible targets for prevention or intervention (22).

Network analysis has been applied to numerous mental disorders in samples of the general population (23), adolescents (24), older adults (25), nurses (26), and firefighters (27) during the COVID-19 pandemic. Due to the nature of their work, fire service recruits must learn how to deal with various emergencies including public health crises such as virus outbreaks. Therefore, fire service recruits may be more prone to fear of emergent viruses including COVID-19 compared to the general population. However, to date, no network model of fear of COVID-19 has been evaluated among fire service recruits during or after any virus pandemic, although understanding the pattern of fear and its network structure is important for developing effective psychosocial interventions for those confronted with ongoing and future virus outbreaks.

This study examined the prevalence and correlates of fear of COVID-19 among fire service recruits following the cessation of China’s dynamic zero-COVID policy. In addition, we explored the network structure of COVID-19 fear and identified symptoms having the strongest relationships with QoL.

## 2. Methods

### 2.1. Study sample

This cross-sectional study was carried out during February 13–16, 2023. Due to risk of contagion and a continuing closed management policy for fire service recruits (8), face-to-face interviews were not conducted in this study. Following other published studies (28, 29), self-report survey data were collected using the WeChat-embedded “Questionnaire Star” software program which is widely used in survey research. Study invitation and data collection forms were linked to a Quick Response code (QR code) that was distributed to all fire service recruits who attended training courses in Beijing (North China) and Sichuan (Southwest China), Guangxi (South China) and Guizhou (Southwest China) provinces via a major social media platform (WeChat) during the study period. Fire service recruits were required to report their health status during the COVID-19 pandemic; therefore, all recruits used WeChat. Fire service recruits who fulfilled the following criteria were included in this study: (1) adult age (18 years or older), (2) ability to speak and understand Chinese, and (3) status as a fire service recruit during the pandemic. Electronic written informed consent was provided by participants on a voluntary and confidential basis. The ethics committee of China Emergency General Hospital approved the study protocol.

### 2.2. Measures

Socio-demographic information was collected. Perceived physical pain and physical fatigue present in the current COVID-19 wave were recorded using self-reported Numeric Rating Scales (NRS) comprising horizontal lines marked with integers from 0 to 10, with “0” and “10” representing “no suffering” and “unbearable suffering,” respectively. NRS are reliable tools for evaluating severity of pain and fatigue (30).

Fear of COVID-19 was measured by the seven-item, self-report Fear of COVID-19 Scale (FCV-19S) (11). The FCV-19 Scale was originally published in English but has been translated and validated in many countries and areas (31), including China (32). FCV-19S items comprise both a physical dimension (i.e., FOC3: Clammy when think about COVID-19; FOC6: Sleep difficulties caused by worry about COVID-19; FOC7: Palpitations when thinking about COVID-19) and a psychological dimension (FOC1: Afraid of COVID-19; FOC2: Uncomfortable to think about COVID-19; FOC4: Afraid of losing life because of COVID-19; FOC5: Nervous when watching news about COVID-19) (33). Each item was rated from 1 (“Strongly Disagree”) to 5 (“Strongly Agree”), with the total scores ranging from 7 to 35. Following previous research (32), we adopted a FCV-19S cut-off value of 16. Hence, participants with a FCV-19S total score of ≥16 were classified as “those with fear of COVID-19,” while those with a FCV-19S total score of <16 were classified as “those with less fear of COVID-19.”

Depression was measured with the nine-item Patient Health Questionnaire (PHQ-9) (34), a self-report measure of depression; items were rated on a 4-point frequency scale and total scores ranged between 0 and 27. Self-reported anxiety was assessed using the seven-item Generalized Anxiety Disorder scale (GAD-7) (35). Each GAD-7 item was rated from 0 (not at all) to 3 (nearly every day); total scores range from 0 to 21. Following previous research (28), the sum of the first two items of another self-report scale, the World Health Organization Quality of Life-brief version (WHOQOL-BREF) (36), was used to measure global quality of life (QoL). Higher total scores reflected better QoL.

### 2.3. Data analysis

#### 2.3.1. Univariate and multivariate analyses

Data analyses were performed using R software (37). Differences in socio-demographic and clinical characteristics of participants “with fear of COVID-19” versus those with “low fear of COVID-19” were assessed using univariate analyses. QoL differences between these subgroups were compared using analysis of covariance (ANCOVA) after controlling for other significant differences identified from univariate analysis. Factors independently associated with fear of COVID-19 status were examined using a binary logistic regression analysis with “Enter” method. The significance level was set at *p* < 0.05 for all tests (two-tailed).

#### 2.3.2. Network structure

Estimation and visualization of the fear of COVID-19 network model were conducted with R-packages “qgraph” (21), “bootnet” (38), the least absolute shrinkage and selection operator (LASSO) and extended Bayesian information criteria (EBIC) (38). Node relationships in green color reflected positive correlations while red color edges reflected negative correlations. To examine the most central nodes, Expected Influence (EI) was adopted as a centrality index (39). R package “mgm” (40) estimated the predictability of each node. Individual fear of COVID-19 symptoms that were directly related to QoL were identified using the “flow” function in R package “qgraph” (21). In addition, 1,000 case-dropping bootstraps were used to estimate stability of the network model, which was graphically represented by calculating the correlation stability coefficient (CS-C).

## 3. Results

### 3.1. Characteristics of the study sample

Of the 1,564 fire service recruits invited to participate, 1,560 (99.74%) met the study selection criteria and were included in analyses. The sample comprised men only with 874 (56.03%) reporting an education level of undergraduate/college degree or above.

### 3.2. Prevalence and correlates of fear of COVID

The overall prevalence of fear of COVID-19 (FCV-19S total score ≥ 16) was 38.85% (*n* = 606; 95% CI = 36.42–41.32%). The fear of COVID-19 mean score for fire service recruits also approached this cut-off (M = 14.52) (SD = 5.64). Table 1 presents demographic data of participants.

**Table 1:** Comparison of demographic and clinical characteristics between fire service recruits with fear of COVID-19 and those with no fear of COVID-19.

Variables	Total (*N* = 1,560)		Without fear of COVID-19 (*N* = 954)		With fear of COVID-19 (*N* = 606)		Univariate analyses		
	*n*	%	*n*	%	*n*	%		*df*	*p*
College and above	874	56.03	551	35.32	323	20.71	2.809	1	0.09
Lifetime smoking	580	37.18	345	22.12	235	15.06	0.976	1	0.323
Drinking past year	516	33.08	299	19.17	217	13.91	3.142	1	0.076

	Mean	SD	Mean	SD	Mean	SD	*t/Z*	*df*	*p*
Age (Years)	22.62	1.87	22.65	1.74	22.57	2.04	1.113	–^a^	0.266
PHQ-9 total	1.74	2.92	1.35	2.48	2.35	3.42	−6.200	–^a^	**<0.001**
GAD-7 total	1.57	4.06	1.23	3.74	2.12	4.48	−5.845	–^a^	**<0.001**
Global quality of life	7.86	1.51	8.13	1.45	7.53	1.53	7.891	–^a^	**<0.001**
Physical pain	1.68	1.94	1.52	1.85	1.93	2.06	−3.856	–^a^	**<0.001**
Physical fatigue	3.11	2.45	2.83	2.41	3.54	2.45	−5.680	–^a^	**<0.001**

a = Mann–Whitney U test; Bolded values are *p* < 0.05; df, degree of freedom; PHQ-9, patient health questionnaire-9 items; GAD-7, generalized anxiety disorder-7 items; SD, standard deviation.

Univariable analyses revealed a statistical trend indicating fire service recruits with fear of COVID-19 were more likely to have a college or above degree (*p* = 0.09). Compared with those who reported lower fear of COVID-19, fire service recruits with fear of COVID-19 had higher mean total PHQ-9 (*p* < 0.001), GAD-7 (*p* < 0.001) perceived physical pain (*p* < 0.001) and perceived physical fatigue (*p* < 0.001) scores in addition to a lower average QoL rating (*p* < 0.001). An ANCOVA found a significantly lower mean QOL score for fire service recruits with fear of COVID-19 compared to those with lower fear of COVID-19 (*F* = 18.061 *p* < 0.001), even after controlling for other significant differences identified from univariate analysis. A binary logistic regression analysis revealed that participants with fear of COVID-19 also reported comparatively more severe depressive symptoms (OR = 1.084; *p* < 0.001) and fatigue (OR = 1.063; *p* = 0.026) (Table 2).

**Table 2:** Independent correlates among fire service recruits during the COVID-19 pandemic (*N* = 1,560).

	*p* value	Odds ratio	95% CI
PHQ-9 total	**<0.001**	1.084	1.038–1.131
GAD-7 total	0.135	1.021	0.994–1.049
Physical pain	0.731	1.011	0.949–1.078
Physical fatigue	**0.026**	1.063	1.007–1.122

Bolded values: <0.05; CI, confidence interval; OR, odds ratio.

### 3.3. Network structure of fear of COVID-19 symptoms

**Figure 1:**
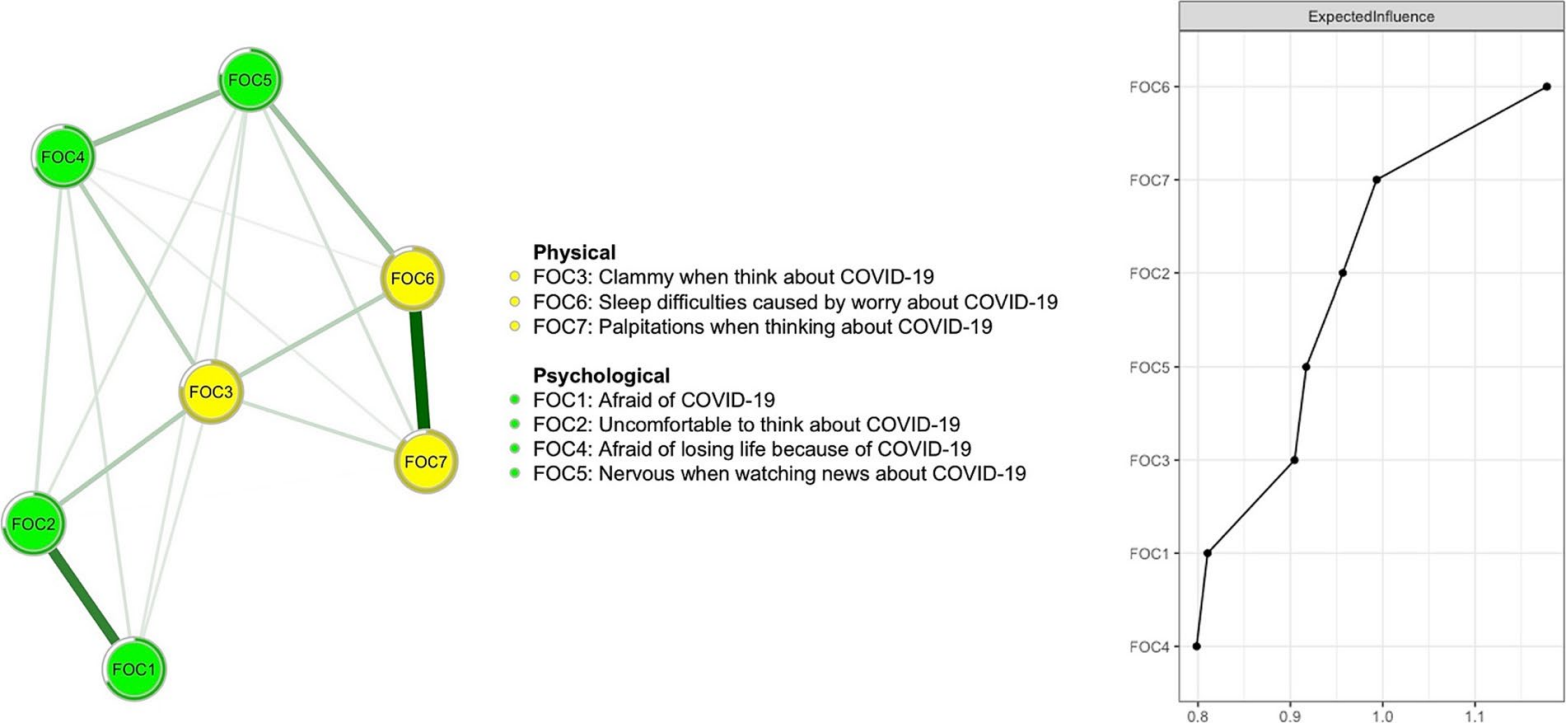
The fear of COVID-19 network structure among fire service recruits during the COVID-19 pandemic.

**Figure 2:**
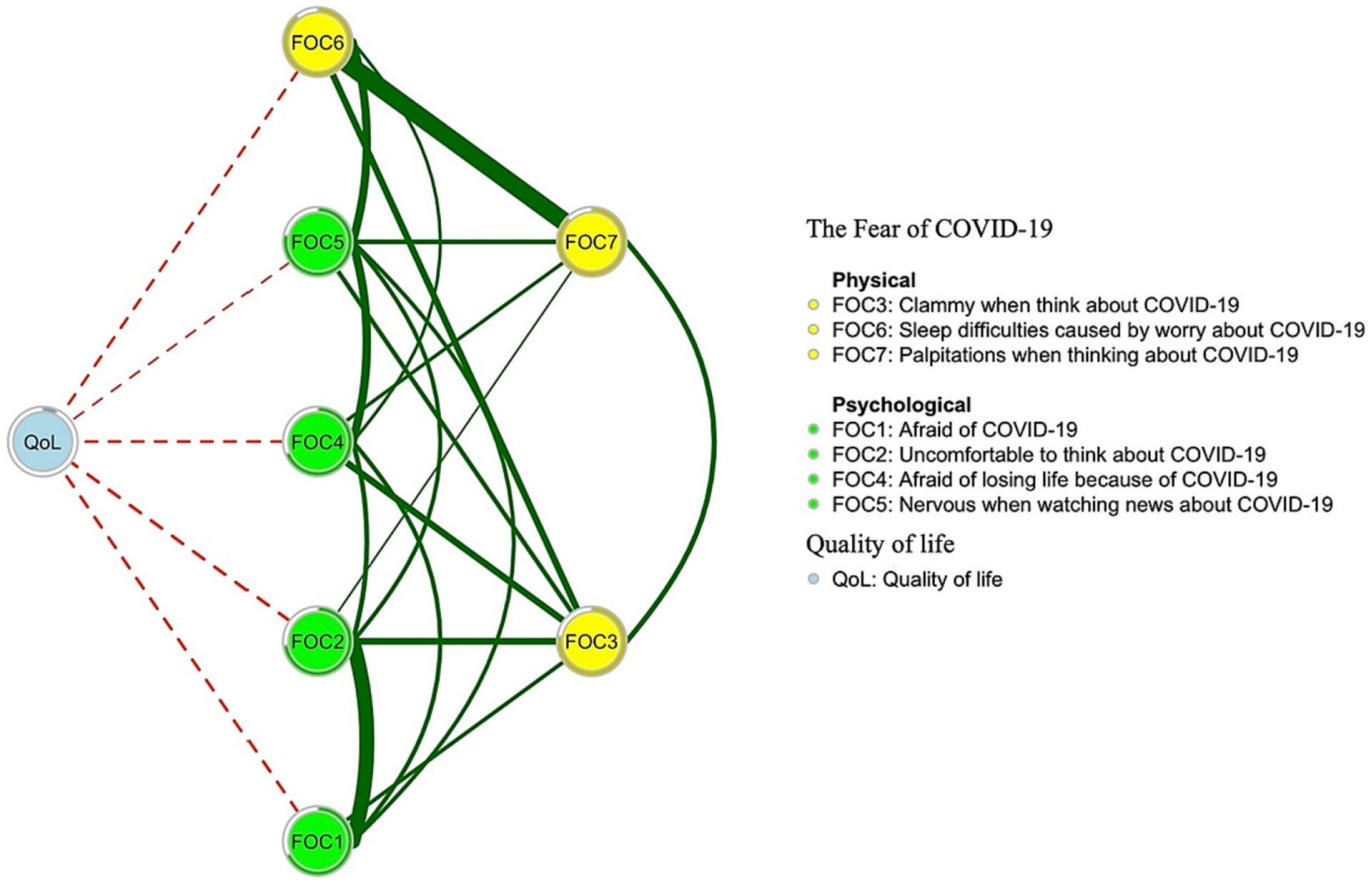
Flow network of quality of life and fear of COVID-19.

Figure 1 shows the network structure for fear of COVID-19 symptoms as measured by FCV-19S items. The three most central symptoms with the highest EI values were nodes FOC6 (“Sleep difficulties caused by worry about COVID-19”) and FOC7 (“Palpitations when thinking about COVID-19”) from the FCV-19S physical dimension, and FOC2 (“Uncomfortable to think about COVID-19”) from the FCV-19S psychological dimension. The mean predictability of nodes was 0.775, which showed that, on average, 77.5% of the variance in each node could be accounted for by other nodes in the network model. Supplementary Table S1 presents network centrality indices for each fear of COVID-19 symptom. Figure 2 shows results of the flow network model. FOC2 (“Uncomfortable to think about COVID-19”; average edge weight = −0.0497) and FOC1 (“Afraid of COVID-19”; average edge weight = −0.0421) from the FCV-19S psychological dimension, and FOC6 (“Sleep difficulties caused by worry about COVID-19”; average edge weight = −0.0314) from the FCV-19S physical dimension had the strongest negative relationships with QoL.

**Figure 3:**
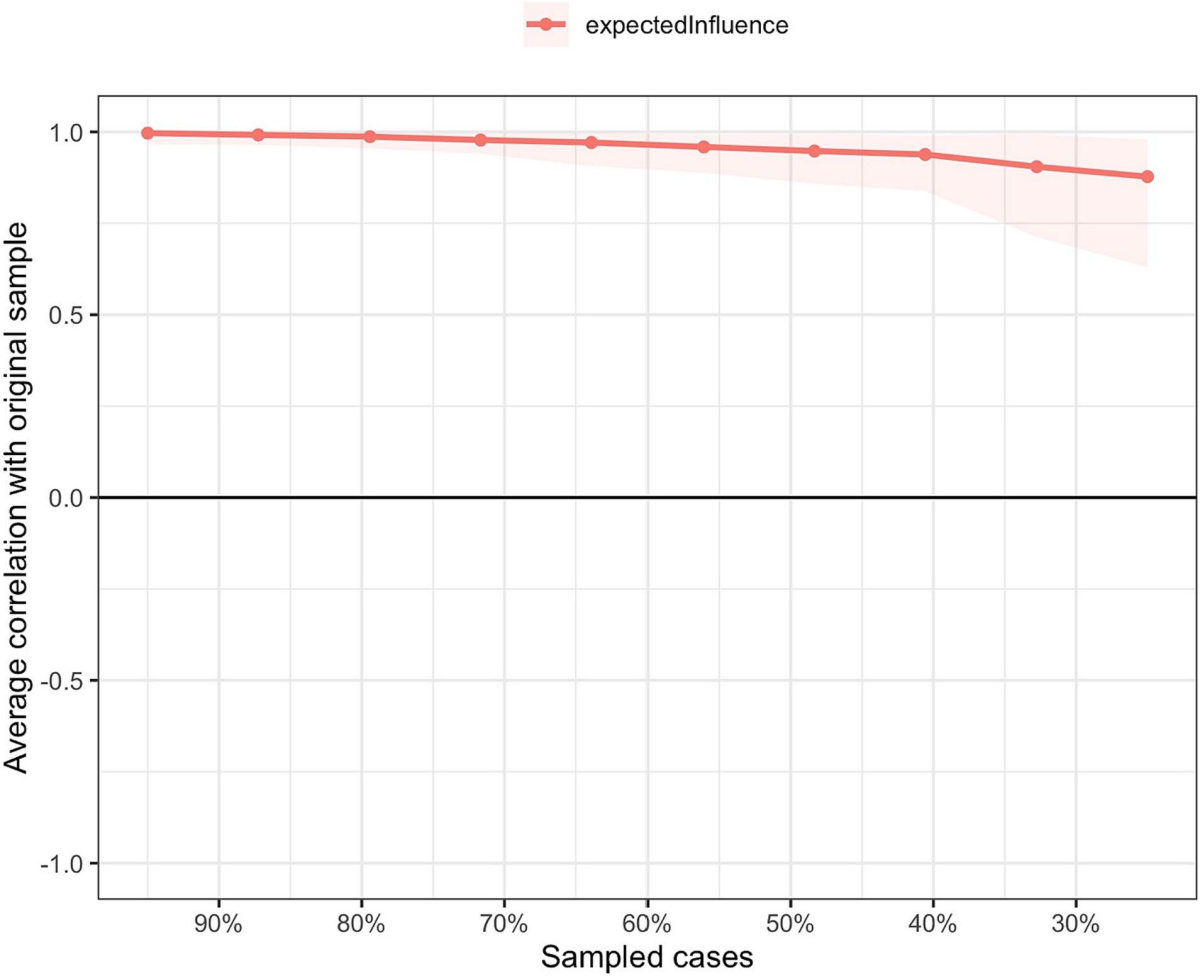
Network stability of the fear of COVID-19 among fire service recruits during the COVID-19 pandemic.

As shown in Figure 3, based on the case-dropping bootstrap procedure, the network model was highly stable. In addition, bootstrap 95% CIs for edge weights used to estimate network accuracy reflected a limited range, with most edge weights being non-zero (Supplementary Figure S1). As such, most edges were stable and accurate. These comparisons were statistically significant, underscoring sound reliability of the network model (Supplementary Figure S2).

## 4. Discussion

To our knowledge, this study is the first to examine the prevalence, correlates, and network structure of COVID-19 fear as well as its relationship with QoL among fire service recruits following the cessation of China’s dynamic zero-COVID policy. The prevalence of COVID-19 fear in this sample was 38.85% (95% CI = 36.42–41.32%). This rate was higher than corresponding figures of 18% among residents in Bosnia and Herzegovina (12), and 33.7% for a general population sample in Lebanon (14), each of which used measures with no clear objective cut-off scores. Conversely, the current rate was lower than the rate of 43.8% found within a Hamburg sample of homeless people, based on a single item COVID-19 fear measure (41). Due to study differences in sample characteristics and assessment methods for examining fear of COVID-19, implications based on direct comparisons between these studies should be drawn with caution. However, in the context of their training during the COVID-19 pandemic, fire service recruits have been required to live in a group environment away from their families for an extended period and face strict training assessments that can cause significant stress (42). Our prevalence results align with the meta-analysis finding of a positive relationship between fear of COVID-19 and stress (43).

We found that more severe depression was associated with a higher level of COVID-19 fear among fire service recruits. After the dynamic zero-COVID policy ceased and a full-blown outbreak followed in China, fire service recruits were trained in a lockdown environment. Similarly, previous studies have found a positive correlation between isolation and depression (44, 45). For example, in a study of Turkish adults, fear of COVID-19 was positively correlated with depression (46). The association between depression and COVID-19 fear may be due, in part, to impaired emotion regulation. People who are depressed may experience more emotional dysregulation (47), which contributes to fear by interfering with the capacity to manage emotions effectively as well as increased sensitivity to perceived threats or stressors (48). A study on fear of COVID-19 and psychiatric comorbidities in the United States also reported significant bivariate associations between fear and symptoms of anxiety and depression (49).

We also found that fire service recruits with fear of COVID-19 were more prone to severe physical fatigue. Fire service recruits must undergo long hours of physical fitness and skill training that may be detrimental to their immediate physical and mental well-being. Fatigue refers to a persistent feeling of tiredness that may affect physical and cognitive performance to the point of increasing fear (50, 51). Previous research (52) has highlighted the interplay between cognitive functions and fear. In addition, when people experience fear of COVID-19, they may show heightened stress responding (53) that exacerbates physical fatigue over time (54).

In the network model of fear of COVID-19, “Sleep difficulties caused by worry about COVID-19” (FOC6) in the FCV-19S physical dimension was the most central symptom. Fire service recruits undergo intense training and testing that can cause psychological stress (6, 8). During the pandemic, recruits may be concerned about their own or others’ health, increasing their stress levels and leading to sleep difficulties or other physical and psychological problems (55). The COVID-19 pandemic has led to global uncertainty (56), so fire service recruits may worry about its effects on their jobs, personal lives, and future career development, leading to anxiety and sleep difficulties (57), that may, in turn, lower QOL. Decreased social support associated with quarantine measures may also contribute to feelings of isolation and sleep disturbances (58). The centrality of “Sleep difficulties caused by worry about COVID-19″ in the network structure highlights potential pandemic effects on mental health of fire service recruits, who are particularly susceptible to pandemic-related stressors.

Another central symptom of COVID-19 fear from the FCV-19S physical dimension was “Palpitations when thinking about COVID-19” (FOC7). While palpitations are commonly associated with cardiovascular diseases, previous studies have found that psychiatric disturbances such as panic, anxiety, depression, and somatoform disorders are frequently observed in patients with palpitations (59). Fire service recruits may experience worry about infecting themselves or others, being unable to perform their job effectively or protecting themselves and others. Palpitations are a psychological and physiological response to perceived threats that often emerge in the context of significant life stressors (60). The centrality of “Palpitations when thinking about COVID-19” suggested that physiological symptoms present in severe anxiety states such as panic (61) warrant attention and may be amenable to interventions based on exposure and learning to realistically reappraise specific physiological reactions (62).

“Uncomfortable to think about COVID-19” (FOC2) from the FCV-19S psychological dimension was another central symptom in the network model among fire service recruits, consistent with findings in a study of Latin American countries (63). Worry and stress about viruses such as COVID-19 may lead to physical reactions such as heart palpitations (61), muscle tension (64), upset stomach (65), and fatigue (54) that contribute to more general experiences of discomfort. To ensure the safety and improve well-being of fire service recruits in the context of virus outbreaks or pandemics, it is important to implement a range of measures that include providing comprehensive training and instruction, establishing safety protocols, offering psychological support and promoting physical fitness.

The top three symptoms that were negatively associated with QoL in the flow network model of QoL and Fear of COVID-19 included “Afraid of losing life because of COVID-19” (FOC4) and “Uncomfortable to think about COVID-19” (FOC2) from the FCV-19S psychological dimension and “Sleep difficulties caused by worry about COVID-19” (FOC6) from the FCV-19S physical dimension. After the dynamic zero-COVID policy ceased, Chinese social media was flooded with news about the pandemic; reports of fever and other symptoms were common thereafter. Consequently, many people, including some fire service recruits, may have experienced increased fear of disability or death from COVID-19. While phobias and anxiety disorders are characterized by excessive and persistent worry, fear and apprehension related to external stimuli or bodily sensations (66), death anxiety pertains specifically to fear and distress associated with death and dying (67). Research has contended that fear of death or death anxiety are common and can become more pronounced in situations where mortality risk is prominent (68). Excessive dread of dying can cause psychiatric illnesses and maladjustment (69) that impair QoL (70). Other possible mechanisms underlying death anxiety (71) include regrets about past events and a lack of meaning in life. Fire service recruits will have demanding, risky, psychologically taxing jobs that require long and arduous training to meet required standards. However, during pandemics, the confinement of their training may cause recruits to question the meaning of their lives and their preparedness to meet job demands. A significant negative association between death anxiety and QoL has been shown in previous studies (72, 73).

Strengths of this study included its assessment of a unique, understudied population, and reliable results from the associated network model. However, the research also had several limitations. First, fear of COVID-19 (FCVS-19) was based on a self-report measure and was susceptible to possible response biases (e.g., social desirability bias and recall biases). Second, since this was a cross-sectional study, causal relations between fear of COVID-19 and other factors cannot be demonstrated. Third, to maintain reasonable assessment burdens on unpaid participants, the survey was highly focused and potentially important influences such as workload, personal life characteristics and use of psychotropic medications were not recorded in this study. Furthermore, potentially salient psychiatric conditions such as post-traumatic stress disorder were not assessed and warrant inclusion in future studies. Fourth, specific sources of COVID-19 related fear (e.g., the virus versus vaccines) could not be determined using the FCV-19S.

## 5. Conclusion

In conclusion, more than one third of fire service recruits reported fear of COVID-19 after China’s dynamic zero-COVID policy was terminated and a full-blown outbreak followed. Increased fear of COVID-19 was associated with other mental health concerns including elevations in depression, anxiety, pain and physical fatigue in addition to diminished QoL. As the most central symptoms in the network structure of COVID-19 fear, “Sleep difficulties caused by worry about COVID-19,” “Palpitations when thinking about COVID-19” and “Uncomfortable to think about COVID-19” appear to be plausible targets for psychosocial interventions among fire service recruits with heightened fear of COVID-19. Based on the core symptoms negatively correlated with QoL (“Afraid of losing life because of COVID-19,” “Sleep difficulties caused by worry about COVID-19” and “Uncomfortable to think about COVID-19”), maintaining physical and mental health during closed training, improving professional training quality and knowledge (74), keeping in touch with family and friends (75), and participating in recreational activities are viable strategies for helping fire service recruits to maintain or improve their QoL (6).

## Data availability statement

The data analyzed in this study is subject to the following licenses/restrictions: the datasets presented in this article are not readily available because The Institutional Review Board (IRB) of the China Emergency General Hospital that approved the study prohibits the authors from making publicly available the research dataset of clinical studies. Requests to access these datasets should be directed to Y-TX, xyutly@gmail.com.

## Ethics statement

The studies involving humans were approved by the ethics committee of China Emergency General Hospital. The studies were conducted in accordance with the local legislation and institutional requirements. The participants provided their written informed consent to participate in this study.

## Author contributions

JL: Conceptualization, Data curation, Formal analysis, Funding acquisition, Writing – review & editing. TS: Data curation, Formal analysis, Methodology, Validation, Writing – original draft, Writing – review & editing. PC: Data curation, Methodology, Writing – review & editing. Y-YW: Data curation, Writing – review & editing. ZS: Data curation, Writing – review & editing. TC: Data curation, Writing – review & editing. TJ: Writing – review & editing. Y-TX: Conceptualization, Funding acquisition, Writing – original draft, Writing – review & editing. YF: Conceptualization, Funding acquisition, Writing – review & editing.

## References

[1] Li W Yang Y Liu ZH Zhao YJ Zhang Q Zhang L. Progression of mental health services during the COVID-19 outbreak in China. Int J Biol Sci. (2020) 16:1732–8. doi: 10.7150/ijbs.45120, PMID: 32226291 PMC7098037

[2] Burki T. Moving away from zero COVID in China. Lancet Respir Med. (2023) 11:132. doi: 10.1016/S2213-2600(22)00508-2, PMID: 36535298 PMC9757876

[3] Ferreira LN Pereira LN da Fé BM Ilchuk K. Quality of life under the COVID-19 quarantine. Qual Life Res. (2021) 30:1389–405. doi: 10.1007/s11136-020-02724-x, PMID: 33389523 PMC7778495

[4] Gostin LO Wiley LF. Governmental public health powers during the COVID-19 pandemic: stay-at-home orders, business closures, and travel restrictions. JAMA. (2020) 323:2137–8. doi: 10.1001/jama.2020.5460 32239184

[5] Lan F-Y Scheibler C Hershey MS Romero-Cabrera JL Gaviola GC Yiannakou I. Effects of a healthy lifestyle intervention and COVID-19-adjusted training curriculum on firefighter recruits. Sci Rep. (2022) 12:10607. doi: 10.1038/s41598-022-10979-2, PMID: 35739126 PMC9226180

[6] Ma HR. Study on the mechanism of combat-oriented training for newly recruited firefighters. China Acad J Electron Publ House. (2020) 24:52–3. doi: 10.16859/j.cnki.cn12-9204/tu.2020.24.031

[7] Chen X Zhang L Peng Z Chen S. Factors influencing the mental health of firefighters in Shantou City, China. Psychol Res Behav Manag. (2020) 13:529–36. doi: 10.2147/PRBM.S249650, PMID: 32753981 PMC7342484

[8] Ministry of Emergency Management of the People's Republic of China. (2022). National Comprehensive Fire Rescue Team 2022 Annual Examination Recruitment Cadre Interview Announcement. Available at: https://www.mem.gov.cn/gk/zfxxgkpt/fdzdgknr/202201/t20220120_407031.shtml (Accessed June 27, 2023).

[9] Tang Y-M Wu T-L Liu H-T. Causal model analysis of the effect of formalism, fear of infection, COVID-19 stress on firefighters’ post-traumatic stress syndrome and insomnia. Int J Environ Res Public Health. (2023) 20:1097. doi: 10.3390/ijerph20021097, PMID: 36673852 PMC9859103

[10] Krueger RF Kotov R Watson D Forbes MK Eaton NR Ruggero CJ. Progress in achieving quantitative classification of psychopathology. World Psychiatry. (2018) 17:282–93. doi: 10.1002/wps.20566, PMID: 30229571 PMC6172695

[11] Ahorsu DK Lin C-Y Imani V Saffari M Griffiths MD Pakpour AH. The fear of COVID-19 scale: development and initial validation. Int J Ment Heal Addict. (2022) 20:1537–45. doi: 10.1007/s11469-020-00270-8 PMC7100496 32226353

[12] Šljivo A Kačamaković M Quraishi I Džubur KA. Fear and depression among residents of Bosnia and Herzegovina during COVID-19 outbreak-internet survey. Psychiatr Danub. (2020) 32:266–72. doi: 10.24869/psyd.2020.266, PMID: 32796797

[13] Meller FO Schäfer AA Quadra MR Demenech LM Paludo SDS da Silva PA. Fear of Covid-19 and health-related outcomes: results from two Brazilian population-based studies. Psychiatry Res. (2022) 313:114596. doi: 10.1016/j.psychres.2022.114596, PMID: 35526424 PMC9065651

[14] Chalhoub Z Koubeissy H Fares Y Abou-Abbas L. Fear and death anxiety in the shadow of COVID-19 among the Lebanese population: a cross-sectional study. PLoS One. (2022) 17:e0270567. doi: 10.1371/journal.pone.0270567, PMID: 35895738 PMC9328531

[15] Usher K Durkin J Bhullar N. The COVID-19 pandemic and mental health impacts. Int J Ment Health Nurs. (2020) 29:315–8. doi: 10.1111/inm.12726, PMID: 32277578 PMC7262128

[16] Salcioglu E Urhan S Pirinccioglu T Aydin S. Anticipatory fear and helplessness predict PTSD and depression in domestic violence survivors. Psychol Trauma. (2017) 9:117–25. doi: 10.1037/tra0000200, PMID: 27710008

[17] Liu RT Kleiman EM Nestor BA Cheek SM. The hopelessness theory of depression: a quarter century in review. Clin Psychol (New York). (2015) 22:345–65. doi: 10.1111/cpsp.12125, PMID: 26709338 PMC4689589

[18] Chrousos G Gold P. The concepts of stress and stress system disorders: overview of behavioral and physical homeostasis, vol. 267. Bethesda, MD: National Institutes of Health (1991). 1244 p. 1538563

[19] Gasparro R Scandurra C Maldonato NM Dolce P Bochicchio V Valletta A. Perceived job insecurity and depressive symptoms among Italian dentists: the moderating role of fear of COVID-19. Int J Environ Res Public Health. (2020) 17:5338. doi: 10.3390/ijerph17155338, PMID: 32722202 PMC7432196

[20] Mertens G Duijndam S Smeets T Lodder P. The latent and item structure of COVID-19 fear: a comparison of four COVID-19 fear questionnaires using SEM and network analyses. J Anxiety Disord. (2021) 81:102415. doi: 10.1016/j.janxdis.2021.102415, PMID: 33962142 PMC8091728

[21] Epskamp S Cramer AO Waldorp LJ Schmittmann VD Borsboom D. Qgraph: network visualizations of relationships in psychometric data. J Stat Softw. (2012) 48:1–18. doi: 10.18637/jss.v048.i04

[22] Cramer AO Van Borkulo CD Giltay EJ Van Der Maas HL Kendler KS Scheffer M. Major depression as a complex dynamic system. PLoS One. (2016) 11:e0167490. doi: 10.1371/journal.pone.0167490, PMID: 27930698 PMC5145163

[23] Si TL Chen P Zhang L Sha S Lam MI Lok KI. Depression and quality of life among Macau residents in the 2022 COVID-19 pandemic wave from the perspective of network analysis. Front Psychol. (2023) 14:1164232. doi: 10.3389/fpsyg.2023.1164232, PMID: 37168423 PMC10165090

[24] Cai H Chow IH Lei S-M Lok GK Su Z Cheung T. Inter-relationships of depressive and anxiety symptoms with suicidality among adolescents: a network perspective. J Affect Disord. (2023) 324:480–8. doi: 10.1016/j.jad.2022.12.093, PMID: 36584712

[25] Jin Y Sun HL Lam SC Su Z Hall BJ Cheung T. Depressive symptoms and gender differences in older adults in Hong Kong during the COVID-19 pandemic: a network analysis approach. Int J Biol Sci. (2022) 18:3934–41. doi: 10.7150/ijbs.69460, PMID: 35844786 PMC9274487

[26] Tokac U Razon S. Nursing professionals' mental well-being and workplace impairment during the COVID-19 crisis: a network analysis. J Nurs Manag. (2021) 29:1653–9. doi: 10.1111/jonm.13285, PMID: 33604981 PMC8014287

[27] Yuan H Ren L Ma Z Li F Liu J Jin Y. Network structure of PTSD symptoms in Chinese male firefighters. Asian J Psychiatr. (2022) 72:103062. doi: 10.1016/j.ajp.2022.103062, PMID: 35339873

[28] Chen P Zhang L Sha S Lam MI Lok KI Chow IHI. Prevalence of insomnia and its association with quality of life among Macau residents shortly after the summer 2022 COVID-19 outbreak: a network analysis perspective. Front Psych. (2023) 14:1113122. doi: 10.3389/fpsyt.2023.1113122, PMID: 36873201 PMC9978518

[29] Cai H Bai W Sha S Zhang L Chow IHI Lei SM. Identification of central symptoms in internet addictions and depression among adolescents in Macau: a network analysis. J Affect Disord. (2022) 302:415–23. doi: 10.1016/j.jad.2022.01.068 35065088

[30] Oldenmenger WH de Raaf PJ de Klerk C van der Rijt CCD. Cut points on 0–10 numeric rating scales for symptoms included in the Edmonton symptom assessment scale in Cancer patients: a systematic review. J Pain Symptom Manag. (2013) 45:1083–93. doi: 10.1016/j.jpainsymman.2012.06.007, PMID: 23017617

[31] Luo F Ghanei Gheshlagh R Dalvand S Saedmoucheshi S Li Q. Systematic review and meta-analysis of fear of COVID-19. Front Psychol. (2021) 12:661078. doi: 10.3389/fpsyg.2021.661078, PMID: 34177712 PMC8231929

[32] Feng Q-y Huang C-w Jia Y-p Liu T Jia H-y Wang K-c. Reliability and validity of the Chinese version of fear of coronavirus disease 2019 scale (Chinese). Acad J Second Mil Univ. (2021) 42:778–82.

[33] Lecuona O Lin C-Y Rozgonjuk D Norekvål TM Iversen MM Mamun MA. A network analysis of the fear of COVID-19 scale (FCV-19S): a large-scale cross-cultural study in Iran, Bangladesh, and Norway. Int J Environ Res Public Health. (2022) 19:6824. doi: 10.3390/ijerph19116824, PMID: 35682405 PMC9180255

[34] Chen M Sheng L Qu S. Diagnostic test of screening depressive disorders in general hospital with the patient health questionnaire. Chin Ment Health J. (2015) 12:241–5.

[35] He X Li C Qian J Cui H Wu W. A study on the reliability and validity of generalized anxiety scale in general hospitals. Shanghai Psychiatry. (2010) 22:200–3.

[36] The WHOQOL Group. Development of the World Health Organization WHOQOL-BREF quality of life assessment. Psychol Med. (1998) 28:551–8. doi: 10.1017/S0033291798006667 9626712

[37] R Core Team. R: a language and environment for statistical computing. R Foundation for Statistical Computing. Vienna, Austria: (2013) Available at: http://www.R-project.org/

[38] Epskamp S Borsboom D Fried EI. Estimating psychological networks and their accuracy: a tutorial paper. Behav Res Methods. (2018) 50:195–212. doi: 10.3758/s13428-017-0862-1, PMID: 28342071 PMC5809547

[39] Robinaugh DJ Millner AJ McNally RJ. Identifying highly influential nodes in the complicated grief network. J Abnorm Psychol. (2016) 125:747–57. doi: 10.1037/abn0000181, PMID: 27505622 PMC5060093

[40] Haslbeck J Waldorp LJ. (2015). Mgm: estimating time-varying mixed graphical models in high-dimensional data. arXiv preprint arXiv:151006871.

[41] Hajek A Bertram F van Rüth V Kretzler B Püschel K Heinrich F. Prevalence and factors associated with fear of COVID-19 among homeless individuals during the COVID-19 pandemic: evidence from the Hamburg survey of homeless individuals. Risk Manag Healthc Policy. (2021) 14:2689–95. doi: 10.2147/RMHP.S317039, PMID: 34194250 PMC8238066

[42] Sun H Yang F Li C-X. Study on the mental health status and social influencing factors of newly recruited firefighters during the intensive training period. China Acad J Electron Publ House. (2022) 41:1155–59.

[43] Şimşir Z Koç H Seki T Griffiths MD. The relationship between fear of COVID-19 and mental health problems: a meta-analysis. Death Stud. (2022) 46:515–23. doi: 10.1080/07481187.2021.1889097, PMID: 33641626

[44] Brooks SK Webster RK Smith LE Woodland L Wessely S Greenberg N. The psychological impact of quarantine and how to reduce it: rapid review of the evidence. Lancet. (2020) 395:912–20. doi: 10.1016/S0140-6736(20)30460-8, PMID: 32112714 PMC7158942

[45] Ruan J Xu Y-M Zhong B-L. Loneliness in older Chinese adults amid the COVID-19 pandemic: prevalence and associated factors. Asia Pac Psychiatry. (2023):e12543. doi: 10.1111/appy.12543 37562972

[46] Satici B Gocet-Tekin E Deniz ME Satici SA. Adaptation of the fear of COVID-19 scale: its association with psychological distress and life satisfaction in Turkey. Int J Ment Heal Addict. (2021) 19:1980–8. doi: 10.1007/s11469-020-00294-0, PMID: 32395095 PMC7207987

[47] Hilt LM Hanson JL Pollak SD. Emotion dysregulation In: Brown BB Prinstein MJ, editors. Encyclopedia of adolescence. San Diego: Academic Press (2011). 160–9.

[48] Berking M Wupperman P. Emotion regulation and mental health: recent findings, current challenges, and future directions. Curr Opin Psychiatry. (2012) 25:128–34. doi: 10.1097/YCO.0b013e3283503669 22262030

[49] Fitzpatrick KM Harris C Drawve G. Fear of COVID-19 and the mental health consequences in America. Psychol Trauma Theory Res Pract Policy. (2020) 12:S17–21. doi: 10.1037/tra0000924 32496100

[50] Tanaka M Tajima S Mizuno K Ishii A Konishi Y Miike T. Frontier studies on fatigue, autonomic nerve dysfunction, and sleep-rhythm disorder. J Physiol Sci. (2015) 65:483–98. doi: 10.1007/s12576-015-0399-y, PMID: 26420687 PMC4621713

[51] Foa EB Kozak MJ. Emotional processing of fear: exposure to corrective information. Psychol Bull. (1986) 99:20–35. doi: 10.1037/0033-2909.99.1.20, PMID: 2871574

[52] Phelps EA LeDoux JE. Contributions of the amygdala to emotion processing: from animal models to human behavior. Neuron. (2005) 48:175–87. doi: 10.1016/j.neuron.2005.09.025, PMID: 16242399

[53] Bakioğlu F Korkmaz O Ercan H. Fear of COVID-19 and positivity: mediating role of intolerance of uncertainty, depression, anxiety, and stress. Int J Ment Heal Addict. (2021) 19:2369–82. doi: 10.1007/s11469-020-00331-y, PMID: 32837421 PMC7255700

[54] Kocalevent RD Hinz A Brähler E Klapp BF. Determinants of fatigue and stress. BMC Res Notes. (2011) 4:238. doi: 10.1186/1756-0500-4-238, PMID: 21774803 PMC3148561

[55] Blix I Birkeland MS Thoresen S. Worry and mental health in the Covid-19 pandemic: vulnerability factors in the general Norwegian population. BMC Public Health. (2021) 21:928. doi: 10.1186/s12889-021-10927-1, PMID: 34001071 PMC8127278

[56] Koffman J Gross J Etkind SN Selman L. Uncertainty and COVID-19: how are we to respond? J R Soc Med. (2020) 113:211–6. doi: 10.1177/0141076820930665, PMID: 32521198 PMC7439590

[57] Wu D Yang T Hall DL Jiao G Huang L Jiao C. COVID-19 uncertainty and sleep: the roles of perceived stress and intolerance of uncertainty during the early stage of the COVID-19 outbreak. BMC Psychiatry. (2021) 21:306. doi: 10.1186/s12888-021-03310-2, PMID: 34126958 PMC8200549

[58] Choi H Irwin MR Cho HJ. Impact of social isolation on behavioral health in elderly: systematic review. World J Psychiatry. (2015) 5:432–8. doi: 10.5498/wjp.v5.i4.432, PMID: 26740935 PMC4694557

[59] Ehlers A Mayou RA Sprigings DC Birkhead J. Psychological and perceptual factors associated with arrhythmias and benign palpitations. Psychosom Med. (2000) 62:693–702. doi: 10.1097/00006842-200009000-00014, PMID: 11020100

[60] Dong M Zheng J. Letter to the editor: headline stress disorder caused by Netnews during the outbreak of COVID-19. Health Expect. (2020) 23:259–60. doi: 10.1111/hex.13055, PMID: 32227627 PMC7104635

[61] Alijaniha F Noorbala A Afsharypuor S Naseri M Fallahi F Mosaddegh M. Relationship between palpitation and mental health. Iran Red Crescent Med J. (2016) 18:e22615. doi: 10.5812/ircmj.22615, PMID: 27247790 PMC4884607

[62] Margraf J Barlow DH Clark DM Telch MJ. Psychological treatment of panic: work in progress on outcome, active ingredients, and follow-up. Behav Res Ther. (1993) 31:1–8. doi: 10.1016/0005-7967(93)90036-T, PMID: 8093337

[63] Caycho-Rodríguez T Ventura-León J Valencia PD Vilca LW Carbajal-León C Reyes-Bossio M. Network analysis of the relationships between conspiracy beliefs towards COVID-19 vaccine and symptoms of fear of COVID-19 in a sample of Latin American countries. Curr Psychol. (2022):1–16. doi: 10.1007/s12144-022-03622-w, PMID: 36090914 PMC9449951

[64] Pluess M Conrad A Wilhelm FH. Muscle tension in generalized anxiety disorder: a critical review of the literature. J Anxiety Disord. (2009) 23:1–11. doi: 10.1016/j.janxdis.2008.03.016, PMID: 18472245

[65] Creed F Craig T Farmer R. Functional abdominal pain, psychiatric illness, and life events. Gut. (1988) 29:235–42. doi: 10.1136/gut.29.2.235, PMID: 3345935 PMC1433289

[66] Craske MG Rauch SL Ursano R Prenoveau J Pine DS Zinbarg RE. What is an anxiety disorder? Focus. (2011) 9:369–88. doi: 10.1176/foc.9.3.foc369 19957279

[67] Lonetto R Templer DI. (1986). Death anxiety. Washington, DC, US: Hemisphere Publishing Corp. p. 7–37.

[68] Greenberg J Pyszczynski T Solomon S Rosenblatt A Veeder M Kirkland S. Evidence for terror management theory II: the effects of mortality salience on reactions to those who threaten or bolster the cultural worldview. J Pers Soc Psychol. (1990) 58:308–18. doi: 10.1037/0022-3514.58.2.308

[69] Sarfraz M Waqas H Ahmed S Rurush-Asencio R Mushtaque I. Cancer-related stigmatization, quality of life, and fear of death among newly diagnosed cancer patients. Omega (Westport). (2022) 003022282211406. doi: 10.1177/00302228221140650 36409065

[70] Thomson L Sheehan KA Meaney C Wnuk S Hawa R Sockalingam S. Prospective study of psychiatric illness as a predictor of weight loss and health related quality of life one year after bariatric surgery. J Psychosom Res. (2016) 86:7–12. doi: 10.1016/j.jpsychores.2016.04.008, PMID: 27302540

[71] Tomer A Eliason G. Toward a comprehensive model of death anxiety. Death Stud. (1996) 20:343–65. doi: 10.1080/07481189608252787, PMID: 10160570

[72] Maddahi ME Khalatbari J Samadzadeh M Amraei M Ahmadi R Keikhafarzaneh M. The study of quality of life and personality traits of NEO five factors concerning death anxiety in Shahed university students. Int J Sci Eng Res. (2011) 2:1–4.

[73] Sherman DW Norman R McSherry CB. A comparison of death anxiety and quality of life of patients with advanced cancer or AIDS and their family caregivers. J Assoc Nurses AIDS Care. (2010) 21:99–112. doi: 10.1016/j.jana.2009.07.007 20006525

[74] Ding Y Du X Li Q Zhang M Zhang Q Tan X. Risk perception of coronavirus disease 2019 (COVID-19) and its related factors among college students in China during quarantine. PLoS One. (2020) 15:e0237626. doi: 10.1371/journal.pone.0237626, PMID: 32790791 PMC7425914

[75] Zhou L Xie R-h Yang X Zhang S Li D Zhang Y. Feasibility and preliminary results of effectiveness of social media-based intervention on the psychological well-being of suspected COVID-19 cases during quarantine. Can J Psychiatr. (2020) 65:736–8. doi: 10.1177/0706743720932041, PMID: 32483978 PMC7502876

