# Supplementary material for: Prevalence of COVID-19 fear and its association with quality of life among fire service recruits after ceasing the dynamic zero-COVID policy in China
Source: Front Public Health. 2023 Oct 6;11:1257943. doi: 10.3389/fpubh.2023.1257943 (PMC10587416; doi:10.3389/fpubh.2023.1257943)
Supplement: Supplementary file 1 [file Data_Sheet_1.docx]

**Supplementary materials**

Table S1. Descriptive information and network centrality indices of the fear of COVID-19

Figure S1. Bootstrapped confidence intervals of edge weights

Figure S2. Estimation of edge weight difference by bootstrapped difference test

Table S1. Descriptive information and network centrality indices of the fear of COIV-19 symptoms

| Item | Item content | Mean (SD) | Prevalence ^a^ | Predictability | EI |
| --- | --- | --- | --- | --- | --- |
| FOC1 | Afraid of COVID-19 | 2.28 (0.980) | 75.77% | 0.681 | 0.810 |
| FOC2 | Uncomfortable to think about COVID-19 | 2.23 (0.966) | 73.27% | 0.730 | 0.957 |
| FOC3 | Clammy when think about COVID-19 | 2.00 (0.864) | 66.22% | 0.775 | 0.905 |
| FOC4 | Afraid of losing life because of COVID-19 | 2.09 (0.975) | 67.37% | 0.692 | 0.798 |
| FOC5 | Nervous when watching news about COVID-19 | 2.05 (0.911) | 67.63% | 0.783 | 0.917 |
| FOC6 | Sleep difficulties caused by worry about COVID-19 | 1.94 (0.846) | 64.30% | 0.890 | 1.178 |
| FOC7 | Palpitations when thinking about COVID-19 | 1.92(0.843) | 63.31% | 0.874 | 0.993 |

Note:

^a^ The prevalence indicates the rate of the presence of each symptom with a score more than 0.

IQR: interquartile range; SD: standard deviation; EI: Expected influence.

Figure S1. Bootstrapped confidence intervals of edge weights


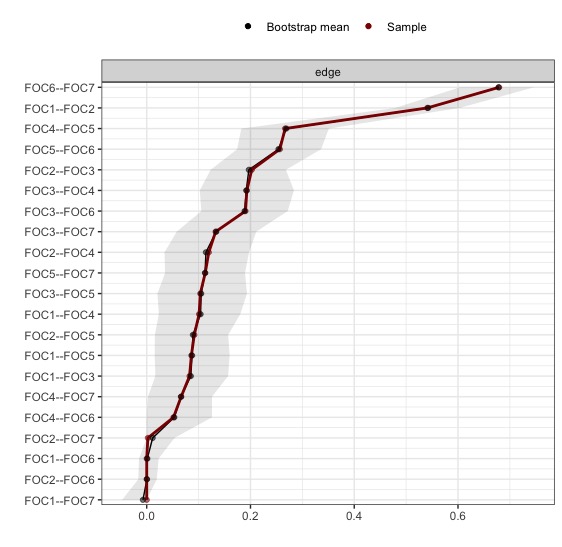


Notes: The black dots indicate the values of each edge weight, ordered from the highest to the lowest value. The gray area represents the 95% confidence intervals of edge weights, estimated with the non-parametric bootstrap procedure. (FOC1: Afraid of COVID-19; FOC2: Uncomfortable to think about COVID-19; FOC3: Clammy when think about COVID-19; FOC4: Afraid of losing life because of COVID-19; FOC5: Nervous when watching news about COVID-19; FOC6: Sleep difficulties caused by worry about COVID-19; FOC7: Palpitations when thinking about COVID-19.)

Figure S2. Estimation of edge weight difference by bootstrapped difference test


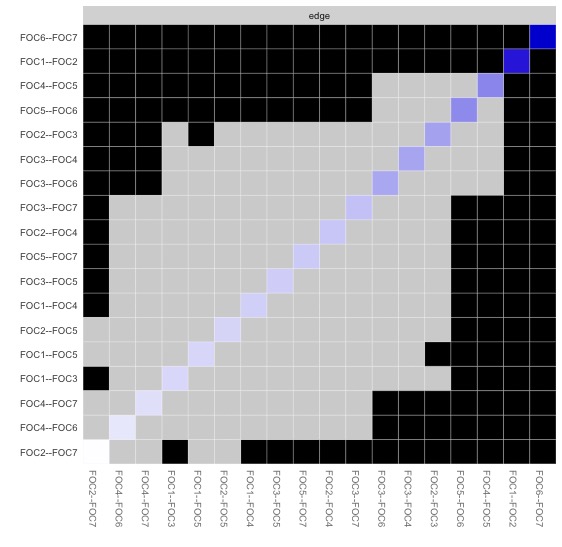


Notes: Gray boxes indicate edges that do not significantly differ from one-another. Black boxes represent edges with significant difference from one another (α = 0.05). Blue boxes in the edge-weight plot indicate positive correlations.
